# Progress of Genomics-Driven Approaches for Sustaining Underutilized Legume Crops in the Post-Genomic Era

**DOI:** 10.3389/fgene.2022.831656

**Published:** 2022-04-07

**Authors:** Uday Chand Jha, Harsh Nayyar, Swarup K Parida, Melike Bakır, Eric J. B. von Wettberg, Kadambot H. M. Siddique

**Affiliations:** ^1^ ICAR-Indian Institute of Pulses Research (IIPR), Kanpur, India; ^2^ Panjab University, Chandigarh, India; ^3^ National Institute of Plant Genome Research (NIPGR), New Delhi, India; ^4^ Department of Agricultural Biotechnology, Faculty of Agriculture, Erciyes University, Kayseri, Turkey; ^5^ Plant and Soil Science and Gund Institute for the Environment, The University of Vermont, Burlington, VT, United States; ^6^ Peter the Great St. Petersburg Polytechnic University, St. Petersburg, Russia; ^7^ The UWA Institute of Agriculture, The University of Western Australia, Perth, WA, Australia

**Keywords:** underutilized legumes, genomics, molecular marker, food security, transcriptomics

## Abstract

Legume crops, belonging to the Fabaceae family, are of immense importance for sustaining global food security. Many legumes are profitable crops for smallholder farmers due to their unique ability to fix atmospheric nitrogen and their intrinsic ability to thrive on marginal land with minimum inputs and low cultivation costs. Recent progress in genomics shows promise for future genetic gains in major grain legumes. Still it remains limited in minor legumes/underutilized legumes, including adzuki bean, cluster bean, horse gram, lathyrus, red clover, urd bean, and winged bean. In the last decade, unprecedented progress in completing genome assemblies of various legume crops and resequencing efforts of large germplasm collections has helped to identify the underlying gene(s) for various traits of breeding importance for enhancing genetic gain and contributing to developing climate-resilient cultivars. This review discusses the progress of genomic resource development, including genome-wide molecular markers, key breakthroughs in genome sequencing, genetic linkage maps, and trait mapping for facilitating yield improvement in underutilized legumes. We focus on 1) the progress in genomic-assisted breeding, 2) the role of whole-genome resequencing, pangenomes for underpinning the novel genomic variants underlying trait gene(s), 3) how adaptive traits of wild underutilized legumes could be harnessed to develop climate-resilient cultivars, 4) the progress and status of functional genomics resources, deciphering the underlying trait candidate genes with putative function in underutilized legumes 5) and prospects of novel breeding technologies, such as speed breeding, genomic selection, and genome editing. We conclude the review by discussing the scope for genomic resources developed in underutilized legumes to enhance their production and play a critical role in achieving the “zero hunger” sustainable development goal by 2030 set by the United Nations.

## Introduction

Burgeoning pressure from the global human population, increasing food demands, and adverse effects of global climate change are serious concerns for global food and nutrition security (Godfray et al., 2010; Foley et al., 2011; Ebi and Loladze 2019). In addition, increasing outbreaks of plant diseases and pests, loss of arable land, and increasing environmental degradation due to excessive use of chemical fertilizers and pesticides have constrained crop yields (Godfray et al., 2010; Lesk et al., 2016). Of the various approaches for sustaining global food production without deteriorating soil and environmental health, crop diversification is needed to maintain sustainable agro-ecological systems and prevent biodiversity losses (Hufnagel J. et al., 2020; Tamburini et al., 2020). Legume crops remain the third most widely grown class of crops globally (Gepts et al., 2005), providing “one third of all dietary protein nitrogen” to the human population, enriching soil fertility by fixing atmospheric nitrogen in association with symbiotically active rhizobacteria in roots (Graham and Vance, 2003), and adding rotational value to subsequent crops (Yigezu et al., 2019; Marques et al., 2020). Likewise, legume fodder and forage mitigate the rising global demand for dietary protein by livestock and provide industrial raw materials (Das and Arora 1978; Elfaki and Abdelatti 2018). Most studies have focused on major grain legumes, such as soybean, common bean, and chickpea. However, some legume crops (Supplementary Table S1) with high nutrient contents are grown in limited areas on small scales in developing countries under low input conditions and marginal land (Cullis and Kunert 2017; Kamenya et al., 2021). Despite the enormous potential of these legumes, they are neglected and known as “underutilized” legumes (Cullis and Kunert 2017; Kamenya et al., 2021). Underutilized species are rarely grown outside of a narrow geographic area, are cultivated with low chemical inputs or mechanization, are not broadly used outside of traditional cuisines, and have not been the focus of major public and private breeding companies. In the last decade, major grain legume crops have witnessed unprecedented advances in genomic resource development, including the development of reference genome sequences due to rapid advances in genome sequencing technologies, especially, next-generation sequencing (NGS). However, underutilized legume crops are lagging behind in terms of developing genomic resources. Thus, in this review we analyze the present global status of these underutilized legumes in terms of area, production, major production and nutritional quality limitation and origin (Supplementary Table S1) and discuss the available genomic resources, including their molecular marker repertoire and genome assemblies. We review the progress in genetic linkage maps and identification of trait QTLs through bi-parental mapping and genome-wide association studies of various underutilized legumes, including the downstream application of genomic assisted breeding (GAB). The discovery of various trait candidate gene(s) with putative function through transcriptome sequencing are discussed with examples. We also brief how crop wild relatives (CWRs), whole-genome resequencing (WGRS), and pangenome sequences could underpin novel structural variants across the whole genome in these crops. Finally, we propose the prospects and scope of novel breeding schemes—genomic selection, genome editing, and speed breeding—for enhancing genetic gain to achieve “zero hunger” in 2030.

### Why Genomics and Advanced Breeding Tools for Underutilized Legumes

Underutilized legumes generally require few inputs, are rich in protein, vitamins, and minerals, and can often withstand harsh environments, including drought, extreme temperature, and waterlogging. Furthermore, these legumes replenish soil nitrogen by fixing atmospheric nitrogen through root nodules, ameliorate soil properties, and sustain agro-ecosystem services (Bhartiya et al., 2015; Ditzler et al., 2021). In addition to their role in combating nutritional and economic security, underutilized legumes play critical roles in various human diseases as they are rich in bioactive compounds and nutraceutical and medicinal properties (Prasad and Singh 2015; Bazzano et al., 2001). However, despite these benefits, there are several constraints and challenges related to the production and productivity of these legumes due to biotic and abiotic stresses (Supplementary Table S1). Furthermore, the edible seeds of some underutilized legumes contain antinutritional elements, constraining their use (Campbell et al., 1994; Tate and Ennenking 2006; Kroc et al., 2017). Combining modern genomic and traditional breeding approaches could help develop new plant types, reduce yield losses from biotic and abiotic stresses, add value for consumer preferences, and eliminate antinutritional properties.

### How Minor Legumes can Catch up With Genomics

One of the aspects of the advances in DNA sequencing technology over the past two decades has been the potential to democratize research. Before the advent of next generation sequencing, performing molecular genetic research outside of a handful of species, such as fruit flies and *Arabidopsis*, was cost-prohibitive. Exponential declines in the cost of sequencing have made research in nearly any species not only feasible, but practical. Consequently, crops like chickpeas, pigeonpea and cowpea, once considered minor crops, now have a rich array of genome resources (e.g., Jha 2018; Varshney et al., 2021). However, there are still a range of crop species that have received less attention, due to being grown over a limited geographic extent or market demand that is mostly restricted to a small region.

For those crop species that still trail behind others for genomic resources, there is hope that lessons learned in other species can be applied to others. In legumes, where there is substantial genome synteny across the entire family [e.g., (Ren et al., 2019)], the potential for comparative genomics to speed up research in understudied species is particularly high. With improving databases for mining genomic information from more widespread cultivated legumes [e.g., (Bauchet et al., 2019; Berendzen et al., 2021)], this task has become easier than in the past.

In a range of minor legume crops, one of the foci for improvement are “domestication syndrome” traits, such as pod shattering, seed dormancy, seed size, and palatability. There is growing evidence that at least some of the loci controlling these traits are shared, such as for pod shattering (Ogutcen et al., 2018). With shared loci and extensive genomic synteny, either finding natural variation at these loci or using genetic modification become much easier.

### Advances in Genomic Resource Development in Underutilized Legumes

In the last decade, rapid advances in genome sequencing technologies have enriched the genomic resources, including genome-wide distributed high-throughput molecular markers especially, simple sequence repeats (SSRs) and single nucleotide polymorphisms (SNPs), transcriptomes, and whole-genome assemblies, of various underutilized legumes.

### Molecular Marker Resources

Hybridization-based molecular markers, such as restriction fragment length polymorphisms (RFLP), and PCR-based molecular markers, such as RAPD, SSR markers, have been used to analyze, tag, and map trait gene(s) in various underutilized legumes (Bohra et al., 2014). However, the arrival of next-generation sequencing technology (NGS) based high-throughput (HTP) markers, especially SNPs, has replaced traditional PCR-based molecular markers for genotyping. Second- and third-generation sequencing technologies have enabled the mining of massive numbers of SSRs and SNPs through whole-genome sequencing, WGRS, and transcriptome sequencing efforts in various crops, including underutilized legumes (Edwards and Batley 2010).

Likewise, the advent of NGS-based HTP genotyping platforms, such as Illumina’s GoldenGate assay, Illumina’s HiSeq 4000 platform, and Illumina’s Infinium SNP array, enabled the discovery of copious SNPs across multiple genomes that facilitate a range of investigations, including the diversity of genebank collections (Sokolkova et al., 2020). Aiming at comprehensive mining of SSR markers for *Vigna* species including cowpea, mungbean and adzuki bean, microsatellite database *VigSatDB* has been developed (Jasrotia et al., 2019). A comprehensive list of molecular markers, mapping populations available in various underutilized legumes are in Table 1. Thus, these molecular markers will provide the foundation for implementing genomic assisted breeding for improving genetic gain in underutilized grain legumes.

**TABLE 1:** Genomic resources in underutilized legumes developed during the last decade.

Common name	Genome size	Mapping populations	SSRs/SNPs discovered
Adzuki bean	538 Mbp (Parida et al., 1990)	∼6 (Yang et al., 2015a; Wang et al., 2021b)	7,947 EST-SSR (Chen et al., 2015a)
—	—	—	143,113 SSRs (Kang et al., 2015)
Bambara	550 Mb(Lonardi et al., 2019)	∼2 (Ho et al. (2017)	1292 SSR (Chapman (2015); 3,343 SNP Uba et al. (2021)
Groundnut	—	—	—
Clusterbean	580.9 Mbp (Tyagi et al., 2019)	—	5,773 (Tanwar et al., 2017); 8,687 (Rawal et al., 2017)
—	—	—	18,792 (Thakur and Randhawa 2018); 25,280 (Kumar et al., 2020)
—	—	—	27,066 (Al-Qurainy et al., 2019); 1,859 genomic SSRs
—	—	—	(Tribhuvan et al., 2019); 3,594 SNPs (Tanwar et al., 2017)
—	—	—	5,999 SNPs and 249 InDels (Thakur and Randhawa 2018)
Common	1.8 Gb (Shirasawa et al., 2021b)	—	6,848 SSRs and 7,246 high quality SNPs (De la Rosa et al., 2020)
Vetch	—	—	—
Dolichos bean	—	∼4 (Konduri et al., 2000; Yuan et al., 2011)	9,320 DArT seq based SNPs and 15,719 SilicoDart markers
or hyacinth bean	—	Ramtekey et al., 2019)	(Sserumaga et al., 2021)
—	—	—	2,529 SSRs (Chapman 2015)
Grasspea	8.2 Gb (Bennett and Leitch, 2012)	∼2 (Santos et al., 2018)	651,827 SSRs and 288 SSRs (Yang et al., 2014); 3,204 EST-SSR
—	—	—	(Hao et al., 2017); 146,406 SNPs (Hao et al., 2017)
Horse gram	400 Mb (Bhardwaj et al., 2013)	∼1 (Shirasawa et al., 2021a)	6,195 SSRs(Bhardwaj et al., 2013)
—	—	—	3,942 SNPs (Mahesh et al., 2021)
Lima bean	∼622 Mbp/1 C	∼1 (Garcia et al., 2021)	10,497 SNPs(Garcia et al., 2021)
—	(Mercado-Ruaro, P. & Delgado-Salinas 1998)	—	—
Mothbean	—	∼1 (Yundeang et al., 2019)	—
Mungbean	494–555 Mb (Liu et al., 2016b)	∼19 (Wang et al., 2020)	13,134 EST-SSRs (Chen et al., 2015b)
—	—	—	and 200,808 SSRs in mungbean
—	—	—	(Kang et al., 2014); 775,831 high-confidence SNPs (Kang et al., 2014)
—	—	—	8,966 SNPs (Ha et al., 2021); 233,799 SNPs (Bangar et al., 2021)
Narrow-	924 Mbp (Kasprzak et al., 2006)	∼9 (Zhou et al., 2018; Kozak et al., 2019)	4830 SNPs (Książkiewicz et al., 2017)
leafed lupin	—	—	38,948 SNPs (Mousavi-Derazmahalleh et al., 2018)
—	—	—	1,723 058 SNPs (Wang P. et al., 2021)
Red clover	420 Mb(Sato et al., 2005)	∼3 (Riday and Krohn 2010)	264,927 SNPs (Jones et al., 2020); 69,975 SNPs (Li et al., 2019)
—	—	—	6,749 SSR, 343,027 SNPs (Ištvánek et al., 2017)
Ricebean	414 Mbp (Kaul et al., 2019)	∼2 (Somta et al., 2006; Isemura et al., 2010)	300 SSR (Chen et al., 2016); 261,458 SSRs (Wang et al., 2016)
White lupin	451 Mb (Hufnagel et al., 2020b)	1	2,659,837 SNPs (Hufnagel et al., 2020b)
—	—	—	3,527,872 SNPs (Hufnagel et al., 2021)
Yellow lupin	—	1 (Iqbal et al. (2020)	13,462 SNPs in yellow lupin (Iqbal et al., 2019)
—	—	—	3,942 SNPs (De Vega et al., 2015)
Urd bean	574 Mbp	3 (Somta et al., 2019)	166,014 SSRs (Jegadeesan et al., 2021)
—	Arumuganathan and Earle (1991)	—	1,621 genic-SSR and 1844 SNPs (Raizada and Souframanien 2019)
—	—	—	3,675 SNPs (Somta et al., 2019)
Winged bean	1.22 Gbp/C (Vatanparast et al., 2016)	—	1853 SSRs (Chapman 2015); 12,956 SSRs(Vatanparast et al., 2016)
—	—	—	5,190 SNPs (Vatanparast et al., 2016)
Zombi pea	—	2 (Dachapak et al., 2018; Amkul et al., 2019)	4,044,822 SNPs in zombipea (Amkul et al., 2019)

### *De Novo* Genome Sequencing of Underutilized Legumes

Adzuki bean (*Vigna angularis* var. *angularis*) (2n = 2x = 22) is an important grain legume of Asiatic origin (Kang et al., 2015). The draft genome sequence of adzuki bean was assembled on 11 pseudo-chromosomes, estimating 612 Mb or 75% of the estimated genome and high-confidence 26,857 protein-coding genes (Kang et al., 2015) (Table 2). Yang K. et al. (2015) assembled a draft genome assembly of “Jingnong 6” cultivar covering 450 Mb of the total genome.

**TABLE 2:** List of genome sequence assembly of underutilised legume crops.

Crop name	Genotype	Pubmed ID	Chromosome no.	Size of genome	No. of protein coding genes	Genome coverage	Sequencing platform used	References
*Cyamopsis tetragonoloba* (L.) Taub.	Vaviloskij 130	—	—	1.2 Gb	—	5×	Illumina and Oxford Nanopore	Grigoreva et al. (2019)
*Cyamopsis tetragonoloba* (L.) Taub.	RGC-936	—	—	550.31 Mbp	34680	366.73×	Illumina, 10× Chromium and Oxford Nanopore	Gaikwad et al. (2020)
*L. purpureus*	—	30535374	2n = 22	395.47 Mb	20,946	—	HiSeq 2000 platform (Illumina)	Chang et al. (2019)
*Lupinus albus*	AMIGA	31980615	2n = 50	451 Mb	38258	164×	PacBio Sequel platform	Bárbara Hufnagel et al. (2020)
*Lupinus angustifolius*	Tanjil	27557478	2n = 40	609 Mb	33 076	162.8×	Illumina	Hane et al. (2017)
*Lupinus angustifolius*	Tanjil	33249667	2n = 40	615.8 Mb	33907	156x	PacBio Sequel II platform	Wang P. et al. (2021)
*Macrotyloma uniflorum*	HPK-4	—	—	259.2 Mb	—	—	Illumina HiSeq 2000	Shirasawa et al. (2021a)
*Macrotyloma uniflorum*	PHG-9	—	2n = 40	279.1 Mb	24,521	—	Illumina HiSeq	Mahesh et al. (2021)
*Medicago polymorpha*	Huaiyang Jinhuacai	33642569	—	441.83 Mb	36,087	123.89×	Illumina, PacBio and Hi-C technologies	Cui et al. (2021)
*M. ruthenica*	—	—	—	904.13 Mb	50,162	—	PacBio, Illumina, 10×Genomics, and Hi-C	Wang T. et al. (2021)
*M. ruthenica*	—	33615703	—	903.56 Mb	50,268	—	Illumina, PacBio, and Hi-C	Mou Yin et al. (2021)
Narrow leafed lupin	Tanjil	23734219	—	538	57,807	27×	—	Yang et al. (2013a)
Narrow leafed lupin	Tanjil	—	—	521.2	—	25×	—	Kamphuis et al. (2015)
*Phaseolus lunatus L*.	G27455	33514713	—	512 Mbp	28,326	10×	Illumina HiSeq	Garcia et al. (2021)
*Phaseolus acutifolis* A. Gray	Frijol Bayo	—	2n = 22	684 Mb	27,538	101.28×	Illumina HiSeq platforms	Moghaddam et al. (2021)
*Phaseolus acutifolis* A. Gray	wild accession	—	2n = 22	676 Mb	27,095	—	Illumina HiSeq platforms	Moghaddam et al. (2021)
—	W6 15578	—	—	—	—	—	—	—
Red clover	Tatra	24500806	—	314.6	47,398	50×	Illumina HiSeq 2000	Ištvánek et al. (2014)
Red clover	Milvus B	26617401	—	309 Mb	40,868	30×	Illumina HiSeq 2000	De Vega et al. (2015)
*T. subterraneum* L.	Daliak	27545089	—	471.8 Mb	42,706	—	Illumina MiSeq and HiSeq 2000	Hirakawa et al. (2016)
*T. subterraneum* L.	TSUd_r1.1	28111887	—	512 Mb	31,272	341×	Illumina HiSeq 2000	Kaur et al. (2017)
*Vigna radiata*	VC 1973A	25384727	2n = 22	579 Mb	22,427	—	Illumina HiSeq 2000, GS FLX +	Kang et al. (2014)
*Vigna radiata*	VC 1973A	34275211	2n = 22	475 Mb	30,958	—	PacBio RS II platform	Ha et al. (2021)
*Vigna angularis*	Jingnong 6	26460024	2n = 22	450 Mb	34,183	168×	HiSeq 2000	Kai Yang et al. (2015)
*Vigna angularis* var. *angularis*	IT213134	25626881	2n = 22	612 Mb	26,857	—	Illumina HiSeq 2000	Kang et al. (2015)
*Vicia sativa*	KSR5	—	2n = 14	1.5 Gb	31,146	146×	HiSeq2000	Shirasawa et al. (2021b)
*Vigna mungo*	Pant U-31	—	2n = 22	475 Mb	18655	—	Illumina and Nanopore sequencing	Jegadeesan et al. (2021)
*Vigna mungo*	Chai Nat 80	—	2n = 22	499 Mb	29,411	21.72×	Illumina HiSeq × Ten	Pootakham et al. (2021)
*V. subterranea*	—	30535374	—	535.05 Mb	31,707	—	HiSeq 2000 platform (Illumina)	Chang et al. (2019)
*Vigna umbellata*	VRB3	—	2n = 22	414 Mb	31276	30×	Illumina and PacBio platform	Kaul et al. (2019)

Bambara groundnut (*Vigna subterranean*) (2n = 2x = 22) is an important legume crop, rich in protein (18–26%), carbohydrate (63%), and fat (6.5%) and having inherent drought tolerance capacity (Shegro et al., 2013). It originated from West Africa and is mainly grown in sub-Saharan areas, especially Nigeria (Olukolu et al., 2012). Chang et al. (2019) assembled the genome sequence of bambara groundnut, with a genome size of ∼535.05 Mb with 31,707 protein-coding genes.

Mungbean (*Vigna radiata*, 2n = 2X = 22) is a warm-season legume crop, originated from India and mostly grown in South and Southeast Asian countries. Kang et al. (2014) first assembled the mungbean genome sequence, estimating 421 Mb or 80% of the total genome size and 22,427 protein-coding genes, with scaffold length 431 Mb and N50 length 35.4 Mb covering 314 Mb. Recently, a mungbean genome sequence was assembled with a total scaffold size of 475 Mb and N50 scaffold value of 5.2 Mb (Ha et al., 2021).

Urdbean (*Vigna mungo*, 2n = 2x = 22), native to Indian subcontinent, mostly grown in South and Southeast Asian countries (Kaewwongwal et al., 2015), is a rich source of dietary protein, vitamins, folate, and iron (Kakati et al., 2010). The genome assembly of Chai Nat 80 cultivar measured 499 Mb with an N50 length of 5.2 Mb (Pootakham et al., 2021). Subsequently, Jegadeesan et al. (2021) assembled a genome assembly of urdbean, measuring 475 Mb or 82% of the genome with scaffold N50 of 1.42 Mb and 42,115 genes with coding sequence.

Cluster bean (*Cyamopsis tetragonoloba*, 2n = 2x = 14), native to west Africa and India, an important commercial legume crop widely grown in India and parts of Africa, contains hetero-polysaccharide called guar gum or galactomannan used extensively in the cosmetic and pharmaceutical industries (Gillett 1958). Gaikwad et al. (2020) assembled the first genome sequence of RGC-936 cultivar, measuring 550.31 Mbp with N50 length of 78.27 Mbps and 34,680 protein-coding genes.

Dolichos bean (*Lablab purpureus*) (2n = 2x = 22) is a versatile legume crop of African origin, rich in seed protein and highly tolerant to various abiotic stresses (Maass et al., 2010). It is mostly cultivated in tropical and sub-tropical regions globally (Maass et al., 2010). The genome assembly of *Lablab purpureus* was constructed recently, with an estimated 395.47 Mb genome size and 20,946 protein-coding genes (Chang et al., 2019).

Grass pea (*Lathyrus sativus*) is a climate-resilient legume of Central Asia and Abyssinia origin, diploid (2n = 2x = 14), cool-season legume species (Kamphuis et al., 2015; Emmrich et al., 2020) primarily grown on the Indian subcontinent and in northern and eastern Africa, including Ethiopia (Kumar et al., 2011). The assembled genome size of EIv1 was measured at 8.12 Gbp with scaffold N50 value of 59.7 kbp and 33,819 high-confidence genes (Kamphuis et al., 2015; Emmrich et al., 2020).

Horsegram [*Macrotyloma uniflorum* (Lam.) Verdc.], native to tropical southern Asia, is a diploid legume (2n = 20, 22) grown in India, Africa, and Australia (Arora and Chandel 1972). The genome sequence of the HPK-4 genotype was assembled on ten pseudomolecules measuring 259.2 Mb or 89% of the total length of the assembled sequence (Shirasawa et al., 2021a). Another genome assembly of accession PHG-9, measuring 279.1 Mb with 24,521 annotated genes has recently been constructed (Mahesh et al., 2021).

Red clover (*Trifolium pratense* L.; Fabaceae, 2n = 2x = 14) is an important forage legume of European origin, with a genome size of 418 Mbp. Ištvánek et al. (2014) completed a *de novo* assembly of the red clover genome, comprising ∼314.6 Mbp.

Likewise, subterranean clover presumed to be originated from Southern Australia, belonging to *Trifolium* genus, is an annual diploid (2n = 2x = 16) pasture legume with 540 Mbps genome size (Kaur et al., 2017). Hirakawa et al. (2016) assembled the genome sequence of *T. subterraneum* L., measuring 471.8 Mb or 85.4% of the whole genome and containing 42,706 protein-coding genes. Subsequently, Kaur et al. (2017) assembled an advanced genome assembly of *T. subterraneum* L., estimating 512 Mb with 31,272 protein-coding genes.

Tepary bean (*Phaseolus acutifolius* A. Gray), native to the Sonoran Desert and a sister species of common bean, is gaining attention due to its inherent capacity for biotic and abiotic stress tolerance (Moghaddam et al., 2021) and important source traits for improving biotic and abiotic stress tolerance in common bean (Moghaddam et al., 2021). A reference genome assembly of cultivated landrace *Frijol Bayo*, possessing inherent heat tolerance, was constructed using Illumina X10 and HiSeq platforms and PACBIO with 101.28× sequence coverage, and measured 512,626,114 bp with 27,538 high-confidence genes (Moghaddam et al., 2021).

White lupin (*Lupinus albus* L. 2n = 50) originated from Mediterranean region, contains high protein content (30–40% whole seed) (Bähr et al., 2014) and can use higher soil phosphorus than other legume crops due to its special “cluster root” structure (Lambers et al., 2013). However, improving yield stability and minimizing anti-nutritional alkaloids in white lupin seed through conventional breeding remains challenging. Hence, to elucidate the function of various trait gene(s) related to quality and quantitative importance, Bárbara Hufnagel et al. (2020) assembled a high-quality genome sequence of white lupin, scaling 451 Mb and 38,258 annotated protein-coding genes.

Likewise, narrow-leafed lupin (*Lupinus angustifolious*) is an important grain legume of Mediterranean origin, enriched with dietary protein (40–45%) and fiber (25–30%) (Lee et al., 2006). Hane et al. (2017) assembled the draft genome sequence of Tanjil cultivar, estimating 609 Mb and 33,076 protein-coding genes. Subsequently, Wang et al. (2021a) constructed an improved genome assembly of Tanjil, measuring 615.8 Mb with contig N50 = 5.65 Mb, using a long-read whole-genome sequencing approach.

Common vetch (*Vicia sativa*, 2n = 14) originated from Near Eastern centre of diversity, is a wild and partially domesticated legume crop with a genome size of 1.8 Gb (Shirasawa et al., 2021b). It is used as silage and hay for livestock feeding. The reference genome assembly has been assembled, spanning 1.5 Gb and 31,146 genes (Shirasawa et al., 2021b).

### Quantitative Trait Mapping Through Bi-parental and Multi-Parental Schemes

As most of the traits with agricultural importance including biotic, abiotic stress tolerance and quality traits are governed by multiple gene(s)/quantitative trait loci (QTL). In order to map these traits various molecular breeding approaches are available to breeders, including family based bi-parental mapping approach, marker-assisted backcrossing. Subsequently, the availability of high-throughput molecular markers has accelerated the precise mapping of various trait QTLs through employing novel molecular breeding schemes including MutMap, multi-parental cross (MAGIC), genome-wide association mapping, genomic selection and QTL seq approach (Meuwissen et al., 2001; Cavanagh et al., 2008; Takagi et al., 2013; Takagi et al., 2015). In underutilized legumes several bi-parental mapping populations based on interspecific and intraspecific crosses have been developed aiming at constructing genetic linkage map and mapping/tagging targeted trait QTLs of agronomic importance (for details Table 3). However, mapping resolution of detected QTLs through bi-parental mapping approach remains low. Therefore, to increase the resolution of trait QTLs novel breeding scheme viz., genome-wide association study (GWAS), nested association mapping and MAGIC has been developed. We believe these approaches could be implemented in underutilized legumes to increase the resolution of trait QTLs.

**TABLE 3:** List of high density genetic maps developed in various underutilised legumes.

Crop	Mapping population	Type of population	Size of LG map	Number of marker/loci assigned	Marker density	References
*Trifolium pratense* L*.*	HR × R130, NS10 × HR, NS10×H17L	BC_1_F_1_	836.6 cM	1804 loci	0.46 cM	Isobe et al. (2009)
—	H17L × R130, 272 × WF1680	—	—	—	—	—
—	pC × pV	—	—	—	—	—
*Vigna mungo*	BC48 × TC2210	—	1,588.7 cM	3,675 SNPs	0.57 cM	Somta et al. (2019)
*Vigna radiata*	*Vigna radiata × V. umbellata*	RIL	1,291.7 cM	538 SNPs	2.40 cM	Mathivathana et al. (2019)
*Vigna angularis*	Ass001 × CWA108	F_2_(150)	1,031.17 cM	1571 SNP	0.67 cM	Kai Yang et al. (2015)
*Vigna angularis*	*Vigna nipponensis*: Yesheng10 ×	RIL,153	1,628.15 cM	2032 SLAF	0.80 cM	Liu et al. (2016a)
—	Jihong9218	—	—	—	—	—
Yellow lupin	Wodjil cultivar × P28213	RIL(154)	—	2,450	—	Iqbal et al. (2020)
Yellow lupin	Wodjil cultivar × P28213	RIL(154)	2,261.3 cM	2,458	2.29 cM	Iqbal et al. (2019)
*Lupinus angustifolius L*	83A:476 × P27255	RIL(87)	2,399 cM	34,574 markers/3,508 loci	—	Zhou et al. (2018)
*Lupinus angustifolius L*	Emir × LAE-1	RIL(92)	3,042 cM	4602 markers	—	Kozak et al. (2019)
*Lupinus angustifolius L*	83A:476 × P27255	RIL(153)	2,500.8 cM	9,972 loci	0.85 cM	Hane et al. (2017)
*Lupinus angustifolius L*	Chittick × Geebung	RIL(185)	781.2 cM	2,315	—	Taylor et al. (2021)
*Phaseolus lunatus L.*	UC 92 ×UC Haskell	RIL	1064 cM	522 loci	2.18 cM	Garcia et al. (2021)
Horsegram	HPK-4 × HPKM-193	F_2_	980 cM	1,263 SNPs	—	Shirasawa et al. (2021a)
*Vigna vexillata*	TVNu 240 × TVNu 1,623	F_2_(198)	1,740.9 cM	6,529	0.27 cM	Amkul et al. (2019)
*Vigna radiata*	*Dahuaye* × *Jilyu 9–1*	RIL	1,060.2 cM	1,946 bin markers	0.54	Wang et al. (2020)
*Vigna angularis*	*Vigna angularis* ×	F_2_(143)	1,365.0 cM	2,904	0.47 cM	Wang et al. (2021b)
—	*V. angularis* var. *nipponensis*	—	—	—	—	—
*Vigna aconitifolia*	TN67 × ICPMO056	F_2_(188)	1,016.8	172	7.34 cM	Yundeng et al. (2019)
Horsegram	HPK4 × HPKM249	RIL(190)	1,423.4 cM	211	9.6 cM	Chahota et al. (2020)
*Vigna radiata*	VC 1973A × V2984	190, RIL	—	1,321	—	Kang et al. (2014)
Lathyrus	BGE008277× BGE023542	103, RIL	724.2 cM	307	2.4 cM	Santos et al. (2018)
*Vigna vexillata*	*V*. *vexillata* (JP235863) ×	F_2_	704.8 cM	262	2.87 cM	Dachapak et al. (2018)
—	wild *V. vexillata* (AusTRCF66514)	—	—	—	—	—
Bamara ground nut	IITA686 × Ankpa4	263 F_2_	1,395.2 cM	223 markers	—	Ho et al. (2017)
—	Tiga Nicuru × DipC	71 F_3_	1,376.7 cM	293 markers	—	—

### Progress in High-Density Genetic Map Development for Trait Quantitative Trait Loci Discovery and Mapping

Initially, morphological-based markers, isozymes, RFLP, amplified fragment length polymorphisms (AFLP), randomly amplified polymorphic DNAs (RAPD), and SSR markers were used to construct preliminary genetic linkage maps in various underutilized legumes [for details, (Bohra et al., 2014)]. However, the increasing ease of developing high-throughput SNP markers derived by GBS, restriction site-associated DNA sequencing (RAD-seq), and whole genome resequencing has facilitated developing highly dense/saturated consensus linkage maps in various underutilized legumes.

Several genetic maps of mungbean based on SSR markers have been developed (Bohra et al., 2014). Later, a genetic map measuring 1,060.2 cM was developed from an intraspecific mapping population (Wang et al., 2020) and a denser genetic map with 1,291.7 cM and harboring 538 SNPs was developed from an interspecific mapping population derived from *Vigna radiata × V. umbellate* cross (Mathivathana et al., 2019) (Table 4).

**TABLE 4:** List of selected QTLs identified in various underutilised legume crops.

Crop	Trait	Mapping population	QTL	LG group	Type of marker	PV%	References
Bambara groundnut	Internode length	IITA686 × Ankpa4, F_2_ 263	One major QTL	LG2	DArTseq markers	33.4	Ho et al. (2017)
—	—	Tiga Nicuru × DipC, F3 71	—	—	—	—	—
*Lupinus angustifolius*	Gray leaf spot	83A:476 × P27255, F_8_ RIL	One major QTL, *LOC109334326*	LG19	Microsatellite fragment	98	Zhou et al. (2021)
—	—	—	*LOC109334327*	—	Length polymorphism	—	—
*Lupinus angustifolius*	Vernalisation	Chittick × Geebung, F_2_ and RIL	*efl, Trimethylguanosine*	LG14	SNP	81.95%	Taylor et al. (2021)
—	—	—	*Synthase1-like (LanTGS1)*	—	—	—	—
—	—	—	*Lup005529.1*	—	—	—	—
*Lupinus albus*	Anthracnose	Kiev×P27174 F8, RIL	*antr04_1,antr05_1,antr04_2, antr05_2*	ALB02, ALB04	SNP	14.6–25	Rychel-Bielska et al.(.2020)
—	—	—	*Lalb_Chr02g0142231*	—	—	—	—
—	—	—	*Lalb_Chr02g0141611*	—	—	—	—
—	—	—	*Lalb_Chr02g0141701*	—	—	—	—
—	—	—	*Lalb_Chr04g0264801*	—	—	—	—
*Lupinus luteous*	Domestication related traits	Wodjil×P28213, RIL(156)	Vernalisation responsiveness locus	YL-21, YL-06	SNP, presence	83%	Iqbal et al. (2020)
—	—	—	Alkaloid content, flower and seed	YL-03 and YL-38	Absence variation	—	—
—	—	—	Colour loci	—	Marker	—	—
*Lupinus luteous*	Anthracnose resistance	Alu*Prot*-CGNA × PI385149	Anthracnose resistance QTL	4, 10, 11, 13, 23	SNP	75–83%	Lichtin et al. (2020)
—	and early flowering	F_2_ (188)	Days to flowering QTL	—	—	—	—
*Macrotyloma*	Drought and yield	HPK4 × HPKM249 (RIL,190)	*qDFW01, qDFW02, qDTM01*	LG1,4,6 7	SSR, RAPD, COS	7.3–55.3%	Chahota et al. (2020)
*uniflorum*	—	—	*qRL01, qNSPP01*	—	—	—	—
*Vigna radiata*	Drought	RIL	58 QTLs	—	SNP	6.40–20	Liu et al. (2017)
*Vigna radiata*	Plant height	VC 1973A × V2984, RIL, 187	*Height4-1, Height5-1*	LG4, 5	SNP	6.2–30	Ha et al. (2021)
—	Flower initiation	—	*FI4-1, FI9-1*	LG4,9	SNP	6.4–24	Ha et al. (2021)
—	No. of branches	—	*Branch3-1*	LG3	SNP	6.4	Ha et al. (2021)
—	No. of nodes	—	*Node4-1, Node11-1*	LG4, 11	SNP	6.3–20	Ha et al. (2021)
—	Synchronous maturity	—	*SPM4-1, SPM7-1*	LG4, 7	SNP	6.8–10.3	Ha et al. (2021)
*Vigna aconitifolia*	*C. chinensis*	TN67× IPCMO056, F2(188)	*qVacBrc2.1* and *qVacBrc5.1*	LG2 and 5	SSR	—	Somta et al. (2018)
*Vigna mungo*	*C*. *maculatus*resistance.	BC48 × TC2210, RIL(150)	*qCm_PDS2*.*1, qCm_AUDPS6.1*	LG2, 6 and 7	SNP	7.28–30%	Somta et al. (2019)
—	—	—	*qCm_AUDPS6.2, qCm_AUDPS7*.*1*	—	—	—	—
—	—	—	*qVmunBr6*.*1* and *qVmunBr6*.*2*	—	—	—	—
*Vigna radiata*	Indented Leaflet	*Dahuaye* × *Jilyu 9–1*	Indented Leaflet QTL	LG3 and LG10	SNP	39.70%	Wang et al. (2020)
—	—	—	—	—	—	and 45.4%	—
*Vigna radiata*	*C. chinensis*	—	*VrPGIP1 and VrPGIP2*	LG5	—	—	Zhang et al. (2021)
*Vigna angularis*	Flowering time	*Vigna nipponensis: Yesheng10* × *Jihong9218*	*Fld3.2* and *Fld3.3, Fld5.1 vs. Fld5., and Fld5.2 vs. Fld5.5*	LG03, LG05	SLAF	66–71%	Mao-Sen Liu et al. (2016)
*Vigna angularis*	*Seed size*	*Vigna angularis* ×	12 seed size related QTLs	LG2, 4,5,6 and 9	Indels	3–22%	Wang et al. (2021b)
—	—	*V. angularis* var*. nipponensis*	—	—	—	—	—
*Vigna aconitifolia*	Domestication related traits	TN67 × ICPMO056 F_2_(188)	50 QTLs related to	LG 1, 2, 4, 7, and 10	SSR	4.26–53.66%	Yundeng et al. (2019)
—	—	—	Domestication related trait	—	—	—	—
*Vigna vexillata*	Domestication related traits	JP235863 × AusTRCF66514	37 QTLs related to	LGs 5, 6, 7, 8, 10 and 11	SSR, RAD-seq	5.9–52%	Dachapak et al. (2018)
—	—	F_2_(139)	Domestication related trait				
*Vigna vexillata*	22 domestication-related traits	*V*. *vexillata* (JP235863) ×	37 QTLs	LG1,2,3,4,5,6,7,8,9	SSR, RAD-seq	upto 52%	Dachapak et al. (2018)
—	—	wild V. vexillata (AusTRCF66514)	—	—	—	—	—
—	—	F_2_(139)	—	—	—	—	—
*Vigna vexillata*	*C*.*chinensis* resistance	TVNu 240 × TVNu 1,623	One major and three minor QTLs	—	SNP	—	Amkul et al. (2019)
—	*C.maculatus* resistance	F_2_(198)	one major and	—	—	—	—
—	—	—	one minor QTLs for *C.maculatus*	—	—	—	—

A comprehensive genetic map of urd bean (*V. mungo*) covering 1,588.7 cM with 3,675 SNPs was developed (Somta et al., 2019). Based on a F_2_ population, Kai Yang et al. (2015) developed an initial genetic map in adzuki bean measuring 1,031.17 cM. Wang et al. (2021b) presented a denser genetic map measuring 1,365.0 cM in adzuki bean (*V angularis*). In zombi pea (*V. vexillata*), a high-density linkage map spanning 1740.9 cM harboring 6,529 SNPs with an average distance of 0.27 cM between markers was developed from an F_2_ mapping population of TVNu 240’ × “TVNu 1,623” (Amkul et al., 2019).

Hane et al. (2017) presented a high-density linkage map of narrow-leafed lupin measuring 2,500.8 cM with 9,972 loci and Iqbal et al. (2019) developed a high-density linkage map of yellow lupin measuring 2,261.3 cM. Santos et al. (2018) developed a genetic map of lathryus covering 724.2 cM with 307 loci. Chahota et al. (2020) presented a genetic map for horse gram measuring 1,423.4 cM with 211 loci (Table4).

The above linkage maps can be used to identify various traits of biotic, abiotic stress tolerance, agronomic, and culinary importance in numerous underutilized legumes. The selected major trait QTLs identified in the last decade based on bi-parental mapping populations are listed in (Table 4). Biotic stress remains the most significant yield stress in underutilized grain legumes globally. The increased availability of genomic resources, especially molecular markers, has identified/tagged various disease-resistant QTLs/gene(s); for example, one major QTL *qCc_PDS6.1* against *Callosobruchus chinensis* (bean weevil) and another QTL *qCm_PDS6.1*against *Callosobruchus maculatus* (cowpea weevil) have been identified (Amkul et al., 2019). Likewise, four major QTLs (*antr04_1*, *antr05_1, antr04_2* and *antr05_2*) controlling anthracnose resistance explaining 14–25% (Rychel-Bielska et al., 2020) of the phenotypic variation in white lupin. Restriction site-associated DNA sequencing derived SNP markers were used as candidate markers for the R gene of phomopsis stem blight disease resistance in narrow-leafed lupin (Yang et al., 2013b). Recently, one major QTL with *LOC109334326, LOC109334327* underlying candidate genes was deciphered for gray leaf spot disease in narrow-leafed lupin (Zhou et al., 2021).

Like biotic stresses, abiotic stresses, particularly drought, causes significant yield losses in underutilized legumes (Liu et al., 2017; Chahota et al., 2020). Several QTLs contributing to drought tolerance have been discovered in mungbean (Liu et al., 2017), and horse gram (Chahota et al., 2020).

Low seed-alkaloid content (<0.02%) is a prime objective of quality improvement in lupin. In lupin the *iucundus* allele is a major gene regulating seed alkaloid content. Several mapping populations have been developed for identifying low alkaloid controlling QTLs and gene(s). Li et al. (2011) identified a microsatellite-anchored fragment length polymorphism-derived PCR marker (lucLi) linked to the low-alkaloid locus *iucundus* (0.9 cM). Likewise, Lin et al. (2009) developed a sequence-specific PCR marker (PauperM1) closely linked (1.4 cM) to the low-alkaloid locus *pauper* in white lupin (*Lupinus albus* L.). Moreover, of five SNP markers co-segregating the *pauper* locus in a set of 140 lupin accessions, the *LAGI01_35805_F1_R1* marker was highly linked with this gene and could be used in low seed alkaloid lupin breeding programs (Rychel and Książkiewicz, 2019). Subsequently, Kroc et al. (2019) developed a co-dominant derived cleaved amplified polymorphic sequence (dCAPS) marker (iuc_RAP2-7) from the *RAP2-7* candidate gene of alkaloid locus *iucundus* responsible for seed alkaloid content in narrow-leafed lupin, which could be used in marker-assisted breeding for low alkaloid content in lupin. Furthermore, fine mapping of this seed alkaloid controlling genomic region unveiled four candidate gene(s)—*LOC109339893, LOC109339862, LOC109339875* and *LOC109339876*—on LG7 in the interval of 20.70–20.89 Mb (Wang et al., 2021a).

### Genome-Wide Association Study Approach for Trait Quantitative Trait Loci Identification With Increased Resolution

GWAS is gaining popularity for uncovering genotype–phenotype associations in various plant species, including underutilized legumes (Huang and Han 2014; Liu and Yan 2019), by establishing the genetic basis of the genotype–phenotype association for the trait of interest in a large panel of diverse accessions based on multiple crossing-over events over the recent demographic history of a taxa (Huang and Han 2014). Due to the unprecedented advances in NGS technology, an increasing repertoire of HTP markers in several underutilized legumes have helped to identify loci associated with aspects of complex trait architecture. GWAS has been assisted by the subsequent availability of genome-wide SNP markers for various traits, including phenological traits, quality/nutritional traits, biotic and abiotic stresses, and yield and yield-related traits, in many underutilized legumes (Plewiński et al., 2020). In narrow-leafed lupin, a GWAS incorporating massive analysis of cDNA ends (MACE) markers in 126 gentoypes uncovered significant MTAs related to flower initiation, maturity, plant height, and yield traits (Plewiński et al., 2020). The underlying candidate genes were *Lup019134, Lup015264, Lup021911,* and *Lup021909* for flower initiation, *Lup015264* and *Lup004734* for maturity, *Medtr1g030750* for plant height, and *Lup021835* and *Lup022535* for yield traits (Plewiński et al., 2020).

GWAS has been used increasingly for dissecting complex QTLs controlling various abiotic stresses in crop plants, including underutilized legumes. To elucidate the underlying genomic regions attributing macro- and micro-nutrients in mungbean seeds, Wu et al. (2020) identified 43 MTAs related to calcium, iron, manganese, phosphorus, sulfur, and zinc using inductively coupled plasma (ICP) spectroscopy and GBS-derived SNPs in a set of 95 global mungbean accessions. The explained phenotypic variation ranged from 1 to 38%. Further, Reddy et al. (2021) used a GBS-based GWAS study to dissect the molecular basis of phosphorus uptake efficiency and phosphorus utilization efficiency in 120 mungbean genotypes. The authors uncovered 116 SNPs in 61 protein-coding genes related to phosphorus uptake efficiency and phosphorus utilization efficiency traits. The significantly associated SNPs explained phenotypic variation ranging from 17 to 20% for total phosphorus utilization (under low phosphorus) and it ranged from 15 to 21% for phosphorus utilization efficiency. Six candidate genes—*VRADI01G04370*, *VRADI05G20860*, *VRADI06G12490, VRADI08G20910, VRADI08G00070* and *VRADI09G09030*—regulating phosphorus uptake efficiency and phosphorus utilization efficiency were deciphered (Reddy et al., 2021).

Recently, recruiting 5,041 SNPs in a minicore collection of 293 mungbean accessions identified four significant MTAs for maturation and hypocotyl color within the *Vradi02g04380* gene on chromosome 2 encoding zinc finger A20 and AN1 domain stress-associated protein (Sokolkova et al., 2020). Despite the popularity of GWAS for elucidating marker-trait associations, it has some drawbacks regarding population structure and low-frequency causal alleles causing false negative results (Korte and Farlow 2013). To minimize and overcome the population structure related problems, artificially designed populations such as MAGIC and nested association mapping, could be used [for details (Alseekh et al., 2021)].

### Crop Wild Relatives and Their Genome Assembly for Exploring Novel Trait Genes in Underutilized Legumes

CWRs, including those of underutilized legumes, are a hidden reservoir of novel trait gene(s), offering scope for broadening genetic diversity in crop breeding programs (Warschefsky et al., 2014; Zhang and Batley, 2020). In the past, during domestication process, several genes associated with adaptive traits conferring abiotic stress tolerance were lost rendering modern cultivated crop plants to adapt poorly under stress condition (Warschefsky et al., 2014; Zhang and Batley, 2020). However, CWRs serve as reservoir of these biotic and abiotic stress adaptive genes. Thus, recapturing these genes from CWRs through introgression and novel breeding tools could facilitate in increasing the fitness of genepool (Burgarella et al., 2019). Several CWRs of underutilized legumes. e.g., *V. nakashimae*, are potential sources of bruchid resistance (Somta et al., 2006) and salinity tolerance (Yoshida et al., 2016) in adzuki bean. Likewise, harnessing bruchid resistance genes/genomic regions from *Vigna radiata* var. *sublobata* can improve bruchid resistance in mungbean (Schafleitner et al., 2016) (Table 5). In urd bean, *V. mungo* var. *silvestris* could be promising for transferring bruchid and mungbean yellow mosaic India virus resistance genes into high-yielding urd bean breeding lines (Souframanien and Gopalakrishna, 2006; Souframanien et al., 2010). Further, the genomic sequences of wild underutilized legumes have been assembled to gain insight into the novel trait genes of CWRs. Whole-genome sequencing of *M. ruthenica* offered novel insights into many genes, including the FHY3/FAR1 gene family conferring higher drought tolerance in cultivated *M. sativa* (Wang et al., 2021c). Mou Yin et al. (2021) advocated evidence for multiple family genes and TF family genes, viz., *C*_*2*_ *H*_*2*_ *, CAMTA* and *NAC* attributing various abiotic stress tolerances through chromosome-scale genome sequencing of *M. ruthenica.* Novel SNP and InDel markers were recovered from genome sequencing of *V. radiata* var. *sublobata*; the wild relative accession TC1966 of mungbean could be useful for exploring biotic and abiotic stress tolerant genomic regions through comparative mapping of cultivated mung bean (Kang et al., 2014). Thus, these CWR genomic resources could be used to develop climate-resilient grain legume cultivars.

**TABLE 5:** List of CWRs source of novel trait gene in various underutilized legumes.

Crop	Wild species	Importance	References
Adzuki bean	*V. nakashimae*	Bruchid resistance	Somta et al. (2006)
Adzuki bean	*V. angularis* var. *nipponensis*	Domestication- and fitness-related traits	Kaga et al. (2008)
Adzuki bean	JP205833 of *V*.*riukiuensis*	*Salinity tolerance*	Yoshida et al. (2016)
—	JP107879 of *V*.*nakashimae*	—	—
Grasspea	*L. articulatus* L. (*IG64782 and IG65197*	*Orobanche crenata*	Abdallah et al. (2021)
—	*IG116989*)	*O. foetida* Poir	—
—	*L. aphaca* L. and *L. ochrus*	—	—
Mungbean	JP 2118749	Bruchid resistance and domestication related traits	Isemura et al. (2012)
Mungbean	*Vigna radiata* var. *sublobata*	Bruchid resistance	Kaewwongwal et al. (2015)
Mungbean	V. radiata var. sublobata TC1966	Bruchid resistance	Schafleitner et al. (2016)
Mungbean	*Vigna umbellata*	Mungbean yellow mosaic virus	Sudha et al. (2015)
Wild vigna	*V. riukiuensis, V. trilobata, V. vexillata*	Salinity tolerance	Yoshida et al. (2020)
—	V. luteola, V. marina	—	—
Urd bean	*V. mungo* var. *silvestris*	Bruchid resistance	Souframanien and Gopalakrishna (2006)
—	—	Mungbean yellow mosaic	Souframanien et al. (2010)
—	—	India virus (MYMIV)	—

### Implications of Genomic-Assisted Breeding in Underutilized Legumes

Current advances in genomic resource development in underutilized legumes have enabled breeders to develop improved cultivars. For example, tagging various traits in narrow-leafed lupin, such as LanFTc1 PCR-based INDEL markers for vernalization responsiveness locus *Ku/Julius* (Nelson et al., 2017; Plewiński et al., 2019; Taylor et al., 2019), InDel2, InDel10, and PhtjM7 for *PhtjR* (Yang et al., 2013b; Yang H. et al., 2015), Anseq3 and Anseq4 for *Lanr1* (Yang et al., 2012), and TP222136 and TP47110 markers for antr04_1/antr05_1 and TP338761 for antr04_2/antr05_2 (anthracnose resistance) (Rychel-Bielska et al., 2020), the iucLi co-dominant marker (Li et al., 2011) and RAP2-7 PCR-based dCAPS marker for major alkaloid content locus *iucundus* (Kroc et al., 2019) are available. Likewise, a diagnostic marker LAGI01_35805_F1_R1 linked to *pauper* locus controlling low alkaloid content in white lupin could be used for practicing MAS of white lupin lines with low-alkaloid content (Rychel and Książkiewicz 2019). Moreover, co-dominant markers linked to the *tardus* (Li et al., 2010) and *lentus* (Li et al., 2012) genes, attributed to low pod shattering, could be of interest for developing zero shattering narrow-leafed lupin using marker-assisted breeding.

**FIGURE 1:**
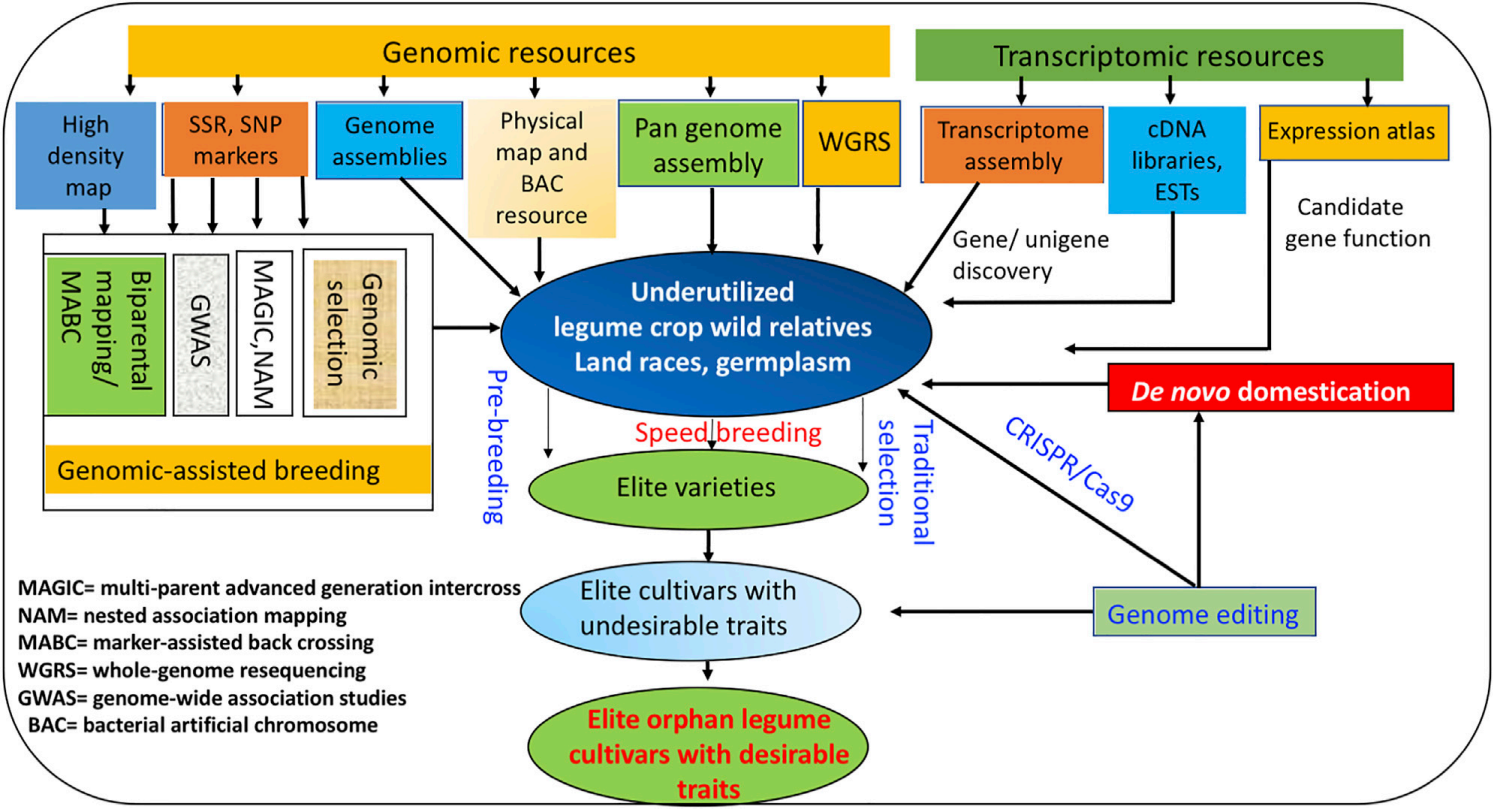
“Omics” and emerging novel breeding approaches for improving genetic gain in underutilized legumes.

Similarly, CEDG261 and DMB-SSR160 markers linked to bruchid resistance could be used in GAB in moth bean breeding programs (Somta et al., 2018). Downstream application of GAB in concert with other novel breeding approaches for enhancing genetic gain in various underutilized legumes is depicted in Figure 1.

### Transcriptomics Resources as a Component of Functional Genomics for Gene Discovery With Function in Underutilized Legumes

The advent of NGS-based RNA-seq technology assessing global gene expression has offered a platform for the discovery of functional markers, including EST-SSRs and SNPs, capturing gene space and shedding light on a myriad of trait candidate genes and their plausible functions (O’Rourke et al., 2013; Yang et al., 2017; Glazińska et al., 2019). Previously, EST markers, microarrays, and cDNA libraries were the major functional genomic resources for investigating the function of various trait genes. For example, cDNA library sequencing identified 125,821 unique sequences (O’Rourke et al., 2013) in white lupin.

Subsequently, advances in transcriptome sequencing facilitated the discovery of many unigenes and differentially expressed genes for various traits of importance for details (see Table 6). Transcriptome studies have also shed light on the functional role of various underlying candidate gene(s) controlling seed biology, plant phenology, biotic and abiotic stress tolerance, yield traits, and nutritional quality traits, including alkaloid regulation in narrow-leafed lupin, β-N-oxalyl-L-α, β-diaminopropionic acid (β-ODAP) in grass pea and condensed tannin in winged bean (Kroc et al., 2019; Yang et al., 2017; Xu et al., 2018).

**TABLE 6:** List of various differentially expressed genes/candidate traits genes with putative function in underutilised legumes.

Crop	Trait	Candidate genes/Unigenes/DEG	Function	Platform used	References
Common vetch	Drought stress tolerance	2,646 transcripts are DEG	Redox homeostasis, cell wall modifications	Illumina HiSeq 2,500	De la Rosa et al. (2020)
Common vetch	Pod shattering	1,285 DEGs and 575 upregulated unigenes	Hydrolase activity	HiSeq 2000	Dong et al. (2017)
—	—	710 downregulated unigenes	Carbohydrate metabolic process	—	—
Guar	Root development	102,479 unigenes	Root development	Illumina HiSeq 2,500	Thakur and Randhawa (2018)
—	stress tolerance	—	Stress tolerance	—	—
Guar	—	11,308	Carbohydrate, protein, lipid, energy	Illumina HiSeq	Tanwar et al. (2017)
—	—	—	Nucleotide metabolism	—	—
Guar	Galactomannan	187 known and 171 novel miRNAs differentially expressed	Regulating galactomannan pathway	Illumina NextSeq 500	Tyagi et al. (2018)
—	Biosynthesis	—	—	—	—
Guar	—	38423 DEGs	Metabolic process, cellular process	Illumina	Rawal et al. (2017)
Guar	Various abiotic stress	61,508 putative genes	Biological process, cellular component and molecular function	Illumina HiSeq 2,500	Al-Qurainy et al. (2019)
Guar	Galactomannan	Cellulose synthase D1, GAUT-like gene	Galactomannan biosynthesis pathway	Illumina HiSeq. 4000	Chaudhury et al. (2019)
—	Biosynthesis pathway	—	—	—	—
Guar—	Galactomannan	5,147 DEGs	*LBD, BZIP, NAC,* and *C2H2, BHLH, MYB*	Illumina Hiseq X Ten	Sharma et al. (2021)
—	Biosynthesis pathway	—	—	—	—
Horsegram	Drought	21,887 unigenes	Calmodulin binding factor, heat shock protein	Illumina GAIIx	Bhardwaj et al. (2013)
—	—	—	DEAD-box ATP dependent RNA helicase	—	—
Lathyrus	Rust tolerance	134,914 contigs	Regulating phytohormone signalling	Illumina Hiseq2000	Almeida et al. (2014)
Lathyrus	*Ascochyta lathyri*	738 unitags	Cell wall metabolism	DeepSuperSAGE	Almeida et al. (2015)
Lathyrus	—	27,431 unigenes	—	llumina NextSeqTM 500	Hao et al. (2017)
Lathyrus	Rust	4520 and 3,498 contigs down regulated	Hormone metabolism, cell wall degradation	Illumina Hiseq2000	Santos et al. (2018)
—	—	—	Secondary metabolism, ROS production	—	—
Lathyrus	β-ODAP	213,258 unigenes	Carbohydrate and	Illumina-HiSeq 3,000	Xu et al. (2018)
—	—	—	sulfur assimilation/metabolism	—	—
—	—	—	nucleic acid metabolism like purine and pyrimidine		—
*Lupinus albus*	Phosphorus	2,128 sequences differentially	Cluster root development	Illumina GA-IIx	O’Rourke et al. (2015)
—	—	expressed in response to Pi deficiency	—	—	—
*Lupinus angustifolius*	—	10,240 transcripts	Peroxidase and anthocyanin biosynthesis	Illumina HiSeq 2000	Kamphuis et al. (2015)
—	—	—	Basal pathogen defences	—	—
*Lupinus angustifolius*	quinolizidine alkaloids	12 candidate genes, RAP2-7, AP2/ERF TF	Quinolizidine synthesis	Illumina HiSeq 1,500	Kroc et al. (2019)
*L. angustifolius*	quinolizidine alkaloids	33 genes related to lupin alkaloid biosynthesis	Copper amine oxidase	Illumina HiSeq 2,500	Yang et al. (2017)
*Medicago ruthenica*	Drought tolerance	3,905 genes and 50 miRNAs	gma-miR171j-5p and mtr-miR396a-5p down regulated	Illumina Hiseq4000	Shi et al. (2021)
Mungbean	MYMV	1881, 1,449, 1,583 and 1,140 genes as up-regulated	Defence related activity	Illumina HiSeq 2,500	Dasgupta et al. (2021)
—	—	1,423, 1,154, 1,396 and 1,152 genes as down-regulated		—	—
Mungbean	Osmotic response	13 *OSCA* genes	Contributes in salinity and drought tress tolerance		Mou Yin et al. (2021)
Mungbean	—	1,245	biological process, cellular component and molecular function	Illumina	Changyou Liu et al. (2016)
*Psophocarpus*	—	5,053 transcript have predicted functions	biological process, cellular component	Illumina platform	Vatanparast et al. (2016)
*tetragonolobus*	—	—	—	—	—
*Trifolium ambiguum*	Rhizome development	betaine aldehyde dehydrogenase	rhizome growth and development	PacBio sequencing	Yin et al. (2020)
—	—	276 DEGs involved in hormone signalling and transduction		and Illumina sequencing	—
*Trifolium pratense*	Drought	45181 contigs	Role of proline, malate and pinitol	Illumina HiSeq 2000 (Illumina, United States)	Yates et al. (2014)
			Contributing to drought tolerance		
*Trifolium pratense*	Seed setting	1,196 DEGs	These gene(s) involved in seed setting	Illumina sequencing platform (HISEQ 2000)	Kovi et al. (2017)
*Trifolium pratense*	Regrowth	Phytohormone related genes	Gibberellin-related genes regulate regrowth in association with other phytohormones	Illumina Hiseq2000	Herbert et al. (2021)
*Trifolium pratense*	Iso-flavonoid	143 iso-flavonoid synthesis genes	Role various genes and long non coding RNAs contributing to iso-flavonoid synthesis	Illumina HiSeq X Ten platform	Shi et al. (2021)
—	—	stem specific genes (*TpPAL, TpC4H*, and *Tp4CL*)	—	—	—
—	—	Root specific genes (*TpCHS*, *TpCHI1*, and *TpIFS*)	—	—	—
*Trifolium repens*	Flower pigmentation	6,282 DEGs, *CHS, F3′H, F3′5′H, UFGT, FLS, LAR, ANS,* and *DFR*)	Anthocyanin flavonoid biosynthetic pathway and flavonoid biosynthetic pathway	Illumina Hiseq ×10	Heshan Zhang et al. (2018)
urd bean	—	2,306 DEGs	Cytochrome c-type biogenesis protein	Illumina MiSeq	Raizada and Souframanien (2019)
—	—	—	DnaJ protein homolog 1	—	
—	—	—	Uncharac- terized protein LOC108329961	—	—
Urdbean	—	29564 transcript contigs	Purine metabolism, pyrimidine metabolism	Illumina	Souframanien and Reddy (2015)
*Vigna angularis*	—	65,950 unigenes	RING-H2 finger protein	—	Chen et al. (2015a)
—	—	—	A serine/threonine protein kinase	—	—
—	—	—	Lipase ROG1-like protein	—	—
*Vigna angularis*	—	324,219 and 280,056 transcripts	—	Illumina HiSeq 2000	Jo et al. (2016)
*Vigna angularis*	Drought	5,337 DEGs	Hormone signal transduction	Illumina HiSeqX	Zhu et al. (2020)
—	—	—	Transcript or translation processes	—	—
—	—	—	Ubiquitin proteasome system	—	—
*Trifolium repens*	Heat stress	*Upregulation of PIP1-1* and *PIP2-7* in leaves	Induction of aquaporin genes	qRT-PCR	Qi et al. (2021)
—	—	and the TIP2-1	Causing heat stress tolerance	—	—
Zombipea	Water logging	982 and 1,133 DEGs	Induction of Cell wall modification	Illumina HiSeq 4000	Butsayawarapat et al. (2019)
—	—	—	Aquaporin, and peroxidase genes	—	—
—	—	—	Auxin Metabolism	—	—

In association with small RNA sequencing, degradome sequencing and transcriptome sequencing helped unravel key molecular players, including various phytohormones and metabolic pathways involved in floral development and organ abscission of *L. luteus* (Glazinska et al., 2017; Glazińska et al., 2019). Moreover, participation of small RNA related to seed biology and the conglutin gene encoding seed storage protein was demonstrated in a transcriptome study in narrow-leafed lupin (DeBoer et al., 2019).

Transcriptome studies could improve our understanding of the regulatory mechanisms of the complex network of gene(s), pathogenesis-related genes, phytohormone signaling response, and non-coding RNAs mediating plant immune responses to attacking pathogens (Almeida et al., 2014; Dasgupta et al., 2021). To gain insight into the molecular mechanisms involved in conferring rust resistance in grasspea, an RNA-seq study in rust-responsive grasspea (resistant vs. susceptible) revealed the upregulation of salicylic acid and abscisic acid in the rust-resistant genotype and downregulation of jasmonate and ethylene pathways in the susceptible genotype (Almeida et al., 2014) (Table 6). Additionally, several pathogenesis-related genes and the mildew resistance locus O (MLO)-like resistance gene were discovered in this study.

An RNA-seq study offered insight into the participatory role of WRKY, NAC and MYB transcription factors, phytoene synthase, cytochrome P450, and JAZ and LOX genes attributing to mungbean yellow mosaic virus (MYMV) resistance (Dasgupta et al., 2021).

Likewise, transcriptome studies can decipher the complex molecular mechanisms and underlying possible candidate gene(s) networks during perceiving abiotic stress signaling and mediate various abiotic stress tolerances by activating antioxidant mechanisms and other cellular protective mechanisms, enabling plants to acclimate to abiotic stress (Bhardwaj et al., 2013; Butsayawarapat et al., 2019; De la Rosa et al., 2020).

A *de novo* transcriptome analysis of two contrasting horse gram genotypes for drought tolerance revealed the involvement of various TFs (NAC, MYB, and WRKY families) in conferring drought stress tolerance (Bhardwaj et al., 2013). De novo transcriptome sequencing of contrasting drought tolerant and sensitive genotypes of common vetch revealed a plethora of differentially expressed genes under water stress (De la Rosa et al., 2020). Most of the genes mediating drought tolerance are associated with cell wall modification, oxidative stress response and ABA response (De la Rosa et al., 2020). In zombi pea, a comparative transcriptome analysis revealed up-regulatory activity of glycolysis and fermentative genes in the waterlogging-sensitive genotype; in contrast, the waterlogging-tolerant genotype had enhanced activity of auxin-regulated lateral root initiation, aquaporin, and peroxidase genes (Butsayawarapat et al., 2019) (Table 6).

Deciphering the underlying genes and molecular function of quality parameters, including nutritional and industrially important parameters, using transcriptomic studies could improve these traits (Yang et al., 2017, Xu et al., 2018; Tyagi et al., 2018). Small RNA sequencing indicated the involvement of several miRNAs and their target genes coding for carbohydrate metabolism, kinase, and enzymes for regulating galactomannan biosynthesis in cluster bean (Tyagi et al., 2018) (Table 6). The authors also discovered two novel unigenes, mannosyltransferase/mannan synthase (ManS) and UDP- D-glucose 4-epimerase (UGE), targeted by Ct-miR3130, Ct-miR3135, and Ct-miR3157 miRNAs. Likewise, an RNA-seq study revealed preferential expression of 2,535 and 2,724 genes in endosperm and 3,720 and 2,530 genes in the embryo involved in guar gum biosynthesis (Hu et al., 2019).

Transcriptome assembly through RNA-seq identified several candidate genes regulating quinolizidine alkaloids (QAs) biosynthesis, an anti-nutritional factor in narrow-leafed lupin (Kamphuis et al., 2015; Yang et al., 2017; Kroc et al., 2019). Short-read sequencing using Illumina HiSeq2500 in association with long-read sequencing using PacBio technology of high QA-containing genotypes identified 33 candidate genes associated with QA biosynthesis in narrow-leafed lupin (Yang et al., 2017). Furthermore, transcriptome profiling offered insight into the genes involved in the accumulation and degradation of β-N-oxalyl-L-α, β-diaminopropionic acid (β-ODAP), a neurotoxin found in grasspea (Xu et al., 2018). Similarly, RNA-seq analysis of high- and low-tannin-containing lines of winged bean, using Illumina Nextseq 500, revealed 1,235 differentially expressed contigs in these two lines. Several genes related to condensed tannin were elucidated, including *anthocyanidin 3-O-glucosyltransferase (A-3GOT), anthocyanidin synthase* (*ANS*)*, chalcone synthase* (*CHS*) *phenylalanine ammonia-lyase (PAL)* (Singh et al., 2017).

### Scope of Genomic Selection/Genomic Prediction for Increasing Genetic Gain in Underutilized Legumes

The decoding of various underutilized legume genome sequences and resequencing efforts have made SNP markers accessible, providing great opportunities to perform genomic selection (GS). This approach has been used for estimating the genomic breeding value of tested individuals without any prior phenotypic information by measuring the genome-wide marker effect based on various prediction models (Meuwissen et al., 2001). Thus, the benefits of GS could be harnessed for the selection of progenies with known genotypic scores with high “genetic merit” for improving genetic gain.

Assessing anthracnose resistance in white lupin using GS based on GBS-derived SNPs in the ridge regression BLUP model, Rychel-Bielska et al. (2020) reported a moderately high predictive ability (0.56). Application of GS is very limited in minor legumes; however, increasing repertoire of genome wide SNP markers will greatly assist in implementing GS for improving future genetic gain in these legumes.

### Scope of Speed Breeding, an Innovative Approach for Enhancing Breeding Efficiency in Underutilized Legumes

Speed breeding could be used to increase breeding efficiency by shortening the breeding cycle and reducing plant space, cost, and labor resources, thereby increasing genetic gain (Watson et al., 2018; Hickey et al., 2019). Speed breeding protocols have been established by optimizing photoperiod, daylength, and temperature in various legume crops, including soybean (Fang et al., 2021), chickpea (Samineni et al., 2019), pigeonpea (Saxena et al., 2019), and pea (Mobini and Warkentin, 2016). However, this approach has not been implemented in any underutilized legumes. Thus, the establishment of a speed breeding protocol could open up new avenues for improving genetic gain in various underutilized legumes more quickly than traditional breeding methods.

### Resequencing and Pangenome Assembly for Capturing Novel Structural Variations Across the Whole Genome

With the declining costs of genome assembly construction, whole genome resequencing is gaining popularity for uncovering genomic regions controlling traits of agronomic importance in a large set of global crop germplasm (Hufnagel B. et al., 2020).

The WGRS approach can elucidate the causal candidate gene(s)/genomic regions associated with traits of interest. Like other major grain legumes, WGRS has been used in underutilized legume crops (Yang H. et al., 2015; Hufnagel B. et al., 2020). The resequencing of nine lupin cultivars discovered 180,596–795,735 SNP markers and 243 candidate diagnostic markers linked to the *PhtjR* (phomopsis stem blight disease) gene (Yang H. et al., 2015). Of these candidate diagnostic markers, nine were validated in commercial cultivars, offering an opportunity to practice marker-assisted breeding for phomopsis stem blight disease resistance in narrow-leafed lupin.

Resequencing 11 modern cultivars, two landraces, and one wild relative of white lupin and comparing them with the reference genome sequence revealed the recent breeding history of white lupin (Hufnagel B. et al., 2020). Similarly, 38 narrow-leafed lupin accessions, including 19 wild and 19 cultivated types, with 19× coverage of the genome were resequenced to reveal the genomic signal for domestication and genes associated with the domestication process (Wang et al., 2021a). A selective sweep analysis in the same study identified 303 genomic regions under strong selection, with 8.2% of the genome under selection associated with domestication. Further, these selective sweeps harbored nine key domestication-related traits, including early flowering, reduced pod shattering, white flower, and low alkaloid (Wang et al., 2021a). WGRS efforts of three mungbean accessions using the Ion Torrent Personal Genome Machine^TM^ (PGM^TM^) platform identified 233,799 SNPs and 9,544 insertions and deletions in coding and non-coding regions, revealing great opportunity for future mung bean improvement using genomic-assisted breeding (Bangar et al., 2021).

Previously, molecular biologists and geneticists have relied mainly on the “single reference genome sequence” of a species for genetic analyses within and across species (Sherman and Salzberg, 2020; Della Coletta et al., 2021). However, the single reference genome sequence does not explain all of the genomic variation/structural variants available within and across species; “pangenomics” studies can capture all of the genomic information in a species. The pangenome refers to the entire non-redundant DNA sequences existing in a species, constituting the “core” genome common to all individuals in a species, with “dispensable” genome the variable fraction or “accessory” genome (Tettelin et al., 2005; Sherman and Salzberg, 2020; Della Coletta et al., 2021; Lei et al., 2021). In the context, Hufnagel et al. (2021) constructed the pangenome of white lupin using a “map to pan” approach (Hu et al., 2017) by sequencing 39 accessions, which identified 32,068 core genes and 14,822 dispensable genes. They also identified 333 selection sweeps related to low alkaloid content and candidate genes (*LaDHDPS, LaHLT,* and *LaAT*) controlling alkaloid content. Pangenome analyses of other underutilized legumes could provide novel insights into genomic variation for future trait discovery.

Several legume genera have multiple domesticated species. For example, *Vigna* has 10 domesticated taxa, *Phaseolus* seven, and *Lupinus* four. Super pan-genomes across these genera might have immense power to provide insight into similarities in domestication syndromes, the genetic basis of traits influencing geographic distribution, and disease and pest resistance.

### Hope and Progress of Genome Editing in Underutilized Grain Legumes

Despite the success of transferring gene(s) of interest into high-yielding cultivars, environmental biosafety and regulatory governing bodies have not allowed the widespread adoption of transgenic technology (Zhang Y. et al., 2018).

Genome editing tools, especially the CRISPR/Cas9 based technique, has revolutionized functional genomics and plant breeding, creating novel genetic variation in plants by editing targeted genes of interest with precision and efficiency (Chen and Gao, 2014). Examples of genome editing in various crops are increasing (Chen and Gao 2014; Zhang Y. et al., 2018); however, there has been limited success in legume species. Notable instances of CRISPR/Cas9 mediated genome editing have been reported in soybean (Cai et al., 2015; Sun et al., 2015; Han et al., 2019), cowpea (Ji et al., 2019) and *Medicago trancatula* (Michno et al., 2015). In case of cowpea, Ji et al. (2019) employed CRISPR/Cas9 based genome editing tool in the symbiosis receptor -like kinase target gene *VuSYMRK* that controls nodule symbiosis in cowpea*.* The edited plant exhibited complete inhibition in nodule formation and consequently, the mutant plants were unable to synthesise nodules in association with *Sinorhizobium* sp. strain NGR234*.* Furthermore, complete male and female sterile plants were generated by editing *SPO11-1* gene through CRISPR/Cas9 technology in cowpea (Juranić et al., 2020). In the context of underutilized legume, the CRISPR/Cas9 genome engineering technique was used to edit the isoflavone synthase gene contributing to rhizobial defense signaling in red clover (Dinkins et al., 2021). Furthermore, gene-editing technology in association with base editors and prime-editing could be harnessed for *de novo* domestication of CWRs of underutilized legumes and “reengineering of metabolism” to increase resilience and enhance nutritive value (Gasparini et al., 2021; Nasti and Voytas 2021).

### Scope of *de Novo* Domestication of Underutilized Legumes

Crop wild relatives are the richest reservoir of genetic diversity for improving various biotic and abiotic stress resistance in crop plants and could therefore be used as new crops through “*de novo* domestication” or “redomestication” process (Fernie and Yan 2019; Von Wettberg et al., 2021). Domestication of new legume underutilized crops from their wild relatives could strengthen crop diversity, and thus be vital for sustainable agriculture (Zhang et al., 2018b). Among the various underutilized grain legume species, *Vigna stipulaceae* could be targeted for *de novo* domestication due to its inherent capacity for drought and salinity stress tolerance and reduced pod shattering (Takahashi et al., 2019). Likewise, being an “incompletely domesticated species” and having inherent stress tolerance ability against biotic and abiotic stress, hairy vetch (*Vicia villosa*) is an ideal legume crop for *de novo* domestication (Renzi et al., 2020).

Of the various approaches, mutagenesis and forward screening and CRISPR/Cas9 based gene editing are important techniques for introducing domestication-related traits in wild relatives for *de novo* domestication (Shapter et al., 2013; Li et al., 2018). Ethyl methanesulfonate mutagenesis and forward screening enabled the domestication of *Vigna stipulacea* Kuntze by selecting mutants with reduced pod shattering and reduced seed dormancy (Takahashi et al., 2019). Likewise, CRISPR/Cas9 genome editing technology could be used to eliminate g-glutamyl-b-cyano-alanine (GBCA) toxin from seeds of common vetch (*Vicia sativa*), providing a zero-toxin vetch variety for combating the rising global protein demand (Nguyen et al., 2020).

## Conclusion and Future Perspectives

Given the rising demand for food, feed, and forage, there is an urgent need to develop sustainable food resources. Underutilized legumes are versatile crops with great potential for mitigating global food security challenges, but they are lagging behind major legumes in terms of genomic resource development. More genomic sequencing of CWRs, landraces, and improved breeding lines will provide novel insights into genomic variations for investigating evolution, domestication events, and the diversification of underutilized legumes. Increasing genomic resources will allow increased genome-assisted breeding of these legumes. Likewise, WGRS in association with GWAS and pangenome integration with GWAS could underpin the causal genes/haplotypes of complex traits of interest. Emerging genome editing techniques could play a critical role in minimizing toxins or negative parameters associated with various nutritional quality traits, such as editing GBCA encoding gene(s) in common vetch, BOAA encoding gene(s) in grasspea, and genes involved in producing QAs in white lupin. These technologies also have great potential for introducing *de novo* domestication in CWRs by removing phenotypically undesired traits in various CWRs of underutilized legumes.

Moreover, genomic selection and speed breeding approaches could enhance genetic gain in underutilized legumes. The rich diversity in these underutilized legumes needs proper collection, conservation, and characterization (Kamenya et al., 2021). Furthermore, the establishment of sound varietal releases and seed distribution systems could play a central role in popularizing these climate-smart underutilized legumes among farmers (Bohra et al., 2020). Disseminating knowledge on the global demand and profitability of these legumes needs strengthening via extension services, especially in developing countries (Kamenya et al., 2021). Hence, collective genomics, novel breeding knowledge, and sound seed system approaches could improve underutilized legume productivity for securing global food security.
