# Supplementary material for: Progress of Genomics-Driven Approaches for Sustaining Underutilized Legume Crops in the Post-Genomic Era
Source: Front Genet. 2022 Apr 7;13:831656. doi: 10.3389/fgene.2022.831656 (PMC9021634; doi:10.3389/fgene.2022.831656)
Supplement: Supplementary file 1 [file Table1.docx]

| **Table1. List of underutilised legume crops and their origin distribution and economic importance** | | | |  |  |  |  |
| --- | --- | --- | --- | --- | --- | --- | --- |
| **Common name** | **Scientific name** | **Origin and distribution** | **Global Production** | **Genome** | **Economic importance** | **Mode of** | **Major production and quality** |
|  |  |  |  | **sequence** | | **pollination** | **Constraints** |
|  |  |  |  | **status** |  |  |  |
| Adzuki bean | *Vigna angularis* | Originated in Northeast Asia (Lee 2013), | The estimated area of adzuki bean in | A | Rich in protein , starch, bioactive compounds and due to low caloric | SP | Adzuki bean mosaic potyvirus (Iizuka,1990) |
|  |  | domesticated in China (Liu et al.2013), | China, Japan, Korean peninsula, and Taiwan |  | and fat content. it is also known as “weight loss bean” |  | bruchids (Vaughan et al., 2005), |
|  |  | cultivated mainly in East Asian countries like | to be 670,000, 120,000, 30,000, and 20,000 ha | | (Amarowicz et al.2008; Kitano-Okada et al.2012) |  | bean common mosaic potyvirus( Iizuka,1990) |
|  |  | China, Japan, and Korea (Kang et al.2015) | (Rubatzky et al.1997) |  |  |  | bean yellow mosaic potyvirus ( Iizuka,1990) |
| Bambara | *Vigna subterranea* (L.) | It is originated from Africa(Hepper, 1963) | Cultivated across the African continent, | NA | It is protein-rich (18–26%), drought tolerant and has | SP | Cowpea mottle virus (Zongo et al.2018) |
| groundnut | Verdc. | Nigeria and Cameroon are the | especially Sub-Saharan Africa and |  | several medicinal benefits (Olukolu et al.2012;Shegro et al.2012) |  |  |
|  |  | origin of dispersal(Cheng et al.2019) | Nigeria as the largest producer in Africa |  |  |  |  |
|  |  |  | (Hillocks et al.2012; |  |  |  |  |
|  |  |  | Mayes t al.2019; Tan et al. 2020) |  |  |  |  |
| Bur clover | *Medicago polymorpha* | Mediterranean Basin (Bounejmate et al 1992), | - | A | Used as palatable forage, | - | *Phoma medicaginis* and |
|  |  | found globally (Wei et al.2015) |  |  | used as fresh and cooked |  | *Leptosphaerulina trifolii* |
|  |  |  |  |  | form in China (Wei et al.2015) |  | (Barbetti et al.2007) |
| Common | *Vicia sativa* | Distributed across | 934,388 tons/year, with a crop | A | It is used as pasture silage and hay (Kim et al.2015) | - | Powdery mildew , Downy Mildew (Soylu et al.2020) |
| Vetch |  | Mediterranean Basin, | area of 540,761 ha (www.fao.org/faostat; |  | It contains 24 to 32% seed protein ( Francis et al., 2000) |  | β-Cyano-L-alanine (Tate and Ennenking,2006) |
|  |  | west and central Asia, China, | De la Rosa et al.2021) |  |  |  |  |
|  |  | eastern Asia, India and the USA | Global area and production are |  |  |  |  |
|  |  | (Tate and Enneking 2006; | 0.6 million ha and 0.9 million tons (FAO, 2017) |  |  |  |  |
|  |  | Kim et al.2015) |  |  |  |  |  |
| Dolichos bean | *Lablab purpureus* | African orgin (Maass et al.2010) | Total cultivated area | A | Contains high seed protein, adapted to diverse | SP | Bruchid (Callosobruchus spp.) , anthracnose |
| or hyacinth bean | |  | in Bangladesh is 48,000 hectares (Maass et al.2010) | | environment (Robotham et al.2017) |  | drought stress (Yao et al.2013); |
|  |  |  | 10,000 hectares in Kenya (Wang et al.2007) |  |  |  | Bean common mosaic necrosis virus |
|  |  |  | 79,000 hectares in India (Vaijayanthi et al.2015) |  |  |  | (Sengooba et al.1997) |
| Grasspea | *Lathyrus sativus* L | It is first domesticated in the Balkan peninsula | Indian subcontinent as well as in northern | A | It is tolerant to drought and flooding | SP/OCP | Anti nutritional factor |
|  |  | (Kislev 1989) | African countries such as Ethiopia (Campbell 1994;  Kumar et al. 2011). |  | (Yadav et al. 2006; Campbell 1997; Emmrich et al.2021) |  | β-Oxalyl-α,β- |
|  |  |  | Total area of production is 1.50 million ha |  |  |  | diaminopropionic acid (ODAP) |
|  |  |  | with annual production of |  |  |  | (Campbell et al.1994) |
|  |  |  | 1.20 million tons (Sammour 2014) |  |  |  | rust (Almeida et al.2014) |
|  |  |  |  |  |  |  | ascochyta blight, orobanche (Vaz Patto et al.2006) |
| Guar | *Cyamopsis* | Western India and parts of Africa, | ~1-1.25 million tons | A | Source of hetero-polysaccharide called guar gum or  galactomannan ( Das and Arora 1978; Tripathy and Das 2013) , |  | Bacterial blight (Ren et al.2014) |
|  | tetragonoloba |  |  |  | also used as fresh vegetable and fodder, contains |  |  |
|  | (L.) Taub. | India, Pakistan, United  States, China, Australia (Gillett 1958) |  |  | 18% protein and 32% dietary fiber (Gillett 1958) |  |  |
| Horse gram | *Macrotyloma uniflorum* | Africa, North Western Himalayan region |  | A | Used as food, fodder and animal feed, contains 16%–30.4% protein | SP | *Colletotrichum dematium*(Sankar et al.2015) |
|  | (Lam.) Verdc. | secondary centre of origin |  |  | (Patel et al., 1995), enriched with lysine and |  | powdery mildew (Parimala et al.2011) |
|  |  | (Arora and Chandel, 1972) |  |  | vitamins (Thirumaran and Kanchana 2000) |  |  |
|  |  |  |  |  | has nutraceutical importance (Prasad et al., 2015 ) |  |  |
| Lima bean | *Phaseolus lunatus L*. | Northern Mexico to northern Argentina | - | A | 20% protein, rich in important amino acids, ( Jones et al.1922) |  | Bean common mosaic virus (Feng et al.2019) |
|  |  | (Cerda-Hurtado et al.2018) |  |  | > 50% carbohydrates, heat and drought tolerant ( Porch et al.2013) | |  |
| Mothbean | *Vigna aconitifolia* | Domesticated in India, Pakistan, | - | NA | It is highly resistant to drought and heat (Tomooka et al.2002); | SP | Yellow mosaic virus |
|  |  | Afghanistan, Sri Lanka, and Myanmar |  |  | it can be grown under 45 °C and annual rainfall of 200–300 mm | | (Yaqoob et al.2015) |
|  |  | (Purseglove 1974) |  |  | (Blink and Jansen 2006); seed are used for |  |  |
|  |  |  |  |  | human consumption (van Oers 1989) |  |  |
| Mungbean | *Vigna radiata* | India remain the origin of mungbean | India remains the world’s largest producer of | A | Mungbean seeds serve as good source of dietary protein and | SP | Anthracnose (Colletotrichum spp.), |
|  |  | (Fuller et al.2007) | mungbean, accounting for over 50% of the global | | possesses higher levels of folate and iron than most other legumes | | mungbean yellow mosaic virus (MYMV) |
|  |  | it is cultivated mostly in South, | annual production (B6 million tons), followed |  | (Keatinge et al.2011) |  | powdery mildew , bruchid,(Nair et al.2019) |
|  |  | East and Southeast Asia | by China and Myanmar (Nair et al.2012) |  |  |  | waterlogging, salinity and heat(Nair et al.2019) |
| Narrow- | *Lupinus angustifolius* L. | Mediterranean region (‘Old World’ lupins) and | NLL cultivation has grown to span more | A | Narrow-leafed lupin kernel possesses | - | Quinolizidine alkaloid (Kroc et al.2017; |
| leafed lupin |  | North and South America (‘New World’ lupins) | than 600 000 hectares in over 20 countries (FAO, 2013) | | 40%– 45% protein and 25%–30% |  | Frick et al.2017), anthracnose |
|  |  | (Drummond et al., 2012); |  |  | dietary fibre, and low fat and |  | (Talhinhas et al.2016), Phomopsis |
|  |  |  |  |  | carbohydrate content (Lee et al.2006) |  | blight (Cowling et al.1987) |
| Red clover | *Trifolium pratense* L | Central Asia, distributed globally and | - | A | It is used as green manure crop and temporary | CP | Clover cancer, Sclerotinia crown and root rot |
|  |  | temperate and subtropical regions (Nichols |  |  | cover crop and used as |  | (Vleugels et al.2013) |
|  |  | et al.2012; Singh et al.2018) |  |  | forage legume in pastoral livestock farming |  |  |
|  |  |  |  |  | systems (De Vega et al.2015) |  |  |
| Ricebean | *Vigna umbellata* | Nepal, Bhutan, northeast India, Myanmar | - | A | Used as fodder, green manure and vegetable | SP | Blister beetle (*Mylabris pustulata*) |
|  |  | China, northern Thailand, |  |  |  |  | (Katoch, 2020) |
|  |  | Laos (Tian et al. 2013) |  |  |  |  |  |
| Subterranean | *Trifolium* | Native to the Mediterranean region, | Grown 29 million hectares worldwide | A | Among the annual forage legumes its contribution remains | SP | Damping-off and root rot |
| clover | *subterraneum* | West Asia and the Atlantic | (Kaur et al 2017) |  | greatest live- stock feed production (McGuire 1985) |  | (*Phytophthora clandestina*, |
|  |  | coast of Western Europe (Kaur et al.2017) |  |  |  |  | and *Pythium irregulare*)(You et al.2021) |
|  |  |  |  |  |  |  |  |
| White lupin | *Lupinus alba* | Mediterranean region, Greece, Western Turkey | 1,610,969 tonnes from 930,717 ha (FAO 2018) | A | High seed protein content 30 and 40% (Bähr et al.2014) |  | Rust (*Uromyces lupinicolus*) and |
|  |  | and southern Balkans (Gladstones et al.1998) | |  | high levels of tocopherols, lowest glycemic index , |  | brown spot (*Pleiochaeta setosa*) |
|  |  | mostly produced in southern Europe |  |  | high dietary fiber content, gluten-free, low oil, and minimal starch | | (Huyghe, 1997; Etheridge and Bateman 1999) |
|  |  | (Gresta et al .2017) |  |  | (Boschin et al.2008; Boschin et al.2011; Fontanari et al. 2012) |  |  |
|  |  |  |  |  | needs low phosphate fertilizers (Lambers et al.2013) |  |  |
| Urd bean | *Vigna mungo* | India (Chandel et al. 1984); | - | A | Contains dietary protein, starch, vitamins and | SP | Leaf crinkle virus, MYMV and bruchids |
|  | [L.] Hepper | South and Southeast Asian countries | India is the largest producer of urd bean |  | mineral elements, containing a high level of folate and iron |  | (Gautam e al.2016; Mishra et al.2018) |
|  |  | including India, | (Kaewwongwal et al., 2015; |  | (Kakati et al.2010 ) |  |  |
|  |  | Bangladesh, Pakistan, Sri Lanka, | Raizada & Souframanien, 2019 ) |  |  |  |  |
|  |  | Myanmar, the Philippines and Thailand |  |  |  |  |  |
| Winged bean | *Psophocarpus* | Southeastern Asia (Basal et al.2020) | - | NA | Possesses proteins (28%–45% proteins), oils(14%–19%), | SP | Leaf spot (Awurum, and Emechebe,2001) |
|  | *tetragonolobus* | India and Papua New Guinea, |  |  | vitamins and carbohydrates (34-40%) |  |  |
|  |  | along with some African countries |  |  | (Kadam et al., 1984; Adegboyega et al., 2019) |  |  |
|  |  |  |  |  | also rich in bioactive compounds having |  |  |
